# Emergence of Carbapenem resistant Gram negative and vancomycin resistant Gram positive organisms in bacteremic isolates of febrile neutropenic patients: A descriptive study

**DOI:** 10.1186/1471-2334-8-80

**Published:** 2008-06-09

**Authors:** Seema Irfan, Faiza Idrees, Vikram Mehraj, Faizah Habib, Salman Adil, Rumina Hasan

**Affiliations:** 1 Department of Pathology & Microbiology, The Aga Khan University, Karachi, Pakistan

## Abstract

**Background:**

This study was conducted to evaluate drug resistance amongst bacteremic isolates of febrile neutropenic patients with particular emphasis on emergence of carbapenem resistant Gram negative bacteria and vancomycin resistant *Enterococcus *species.

**Methods:**

A descriptive study was performed by reviewing the blood culture reports from febrile neutropenic patients during the two study periods i.e., 1999–00 and 2001–06. Blood cultures were performed using BACTEC 9240 automated system. Isolates were identified and antibiotic sensitivities were done using standard microbiological procedures.

**Results:**

Seven twenty six febrile neutropenic patients were admitted during the study period. A total of 5840 blood cultures were received, off these 1048 (18%) were culture positive. Amongst these, 557 (53%) grew Gram positive bacteria, 442 (42%) grew Gram negative bacteria, 43 (4%) fungi and 6 (1%) anaerobes. Sixty (5.7%) out of 1048 positive blood cultures were polymicrobial. In the Gram negative bacteria, *Enterobacteriaceae *was the predominant group; *E. coli *was the most frequently isolated organism in both study periods. Amongst non- Enterobacteriaceae group, *Pseudomonas aeruginosa *was the commonest organism isolated during first study period followed by *Acinetobacter *spp. However, during the second period *Acinetobacter *species was the most frequent pathogen.

*Enterobacteriaceae *group showed higher statistically significant resistance in the second study period against ceftriaxone, quinolone and piperacillin/tazobactam, whilst no resistance observed against imipenem/meropenem. The susceptibility pattern of *Acinetobacter *species shifted from sensitive to highly resistant one with significant p values against ceftriaxone, quinolone, piperacillin/tazobactam and imipenem/meropenem. Amongst Gram positive bacteria, MRSA isolation rate remained static, vancomycin resistant *Enterococcus *species emerged in second study period while no *Staphylococcus *species resistant to vancomycin was noted.

**Conclusion:**

This rising trend of highly resistant organisms stresses the increasing importance of continuous surveillance system and stewardship of antibiotics as strategies in the overall management of patients with febrile neutropenia.

## Background

Febrile neutropenia is associated with high mortality rate therefore institution of timely and appropriate empirical antibiotic therapy is absolutely essential [1,2]. Globally significant change in the spectrum of organisms and their susceptibility pattern is observed in febrile neutropenic over the past few decades. *Staphylococcus aureus *was the most frequent isolate from these patients in 1950s and early 1960s and was later replaced by Gram-negative bacilli including *Escherichia coli*, *Klebsiella *species and *Pseudomonas aeruginosa *[3]. However since 1980s, resurgence of Gram-positive organisms in this population is evident [4]. Recently non-fermenter Gram negative rods such as *Acinetobacter *species have emerged as pathogens in these patients [5]. Additionally use of broad spectrum antibiotics has resulted in emergence of multi drug resistant Gram negative and gram positive bacteria. Therefore, the choice of empiric therapy should vary according to locally prevalent isolates and their resistance patterns. Currently, use of carbapenem as an empirical monotherapy for febrile neutropenic patient is justified in most centers due to growing resistance against other beta lactam antibiotics [6,7]. Similarly empirical use of vancomycin for suspected penicillin and methicillin resistant Gram positive bacterial infection is recommended [2].

Emergence and spread of carbapenem resistant Gram negative rods is a great concern, especially in a resource limited country such as Pakistan, where treatment alternatives are either unavailable or expensive/toxic with poor outcome [8]. The Aga Khan University is a major tertiary care hospital in Pakistan that caters a large population of severely immuno-suppressed patients. The hospital has a hematology-oncology unit along with a bone marrow transplant unit. Recent reports from our center have documented emergence and spread of carbapenem resistance among multi-resistant non enterobacteriaceae including *Acinetobacter *species and *Pseudomonas aeruginosa *[9,10]. In addition, our hospital infection control surveillance committee also reported rising trend of carbapenem resistance in *Acinetobacter *species and *Pseudomonas aeruginosa*, beginning from the year 2001. This has raised the concern about possibility of emergence of these pathogens in febrile neutropenic patients. Similarly rising trend of methicillin resistant *Staphylococcus aureus *and emergence of vancomycin resistant enterococci amongst the hospitalized patients are of great concern [11]. We therefore conducted a study to evaluate drug resistance in bacteremic isolates of febrile neutropenic patients with particular emphasis on emergence of carbapenem resistant gram negative bacteria. In addition to that we tried to evaluate the frequency of methicillin resistant *Staphylococcus aureus *and vancomycin resistance *Enterococcus *species isolated from blood cultures in these patients.

## Methods

This study was conducted in the department of Pathology and Microbiology, Aga Khan University Karachi, Pakistan. Medical records and blood cultures of febrile neutropenic patients, admitted from year 1999 to 2006 were reviewed. To evaluate the changing pattern and increasing antimicrobial resistance of microorganisms in febrile neutropenic patients, we divided the study duration in two periods, the first period comprised of January 1999 to December 2000 while the second period was from January 2001 to December 2006.

Neutropenia was defined as neutrophil counts of 500 or 1000 with predicted decline to 500. Fever was defined as oral temperature of 38°C or above for at least one hour [1]. Blood cultures received from all febrile neutropenic patients between this six and half year study period were included in the study. To avoid error the duplicates were removed (we considered duplicate if same isolate grew more than once during that admission).

Blood cultures were performed using BACTEC 9240 automated system. A set of aerobic and anaerobic bottles containing brain heart infusion and thioglycolate broth respectively, were used for cultures. Negative blood culture bottles were incubated for seven days before being reporting negative. Blood culture isolates were identified using standard microbiological procedure and further confirmation was done using commercially available API strips [12]. Antibiotic sensitivities of all isolates except non enterobacteriaceae were carried out using Kirby Bauer method [13]. Sensitivity testing for non enterobacteriaceae was done using agar dilution method as recommended by CLSI [13].

Statistical analysis: Descriptive analysis was done by calculating frequency and percentages. In order to evaluate the difference in the sensitivity patterns of both gram negative and gram positive organisms during the two halves of the study period, chi square test or Fisher exact where appropriate was performed with a significance level of 5%.

## Results

During the study period (1999–2006), 726 febrile neutropenic patients were admitted. We reviewed medical records of randomly selected 134/726 (18.4%) patients. Amongst these, 49% belonged to hematological malignancy, 44% had solid organ cancer while in 7% of cases diagnosis was not established. Chest was the commonest site of infection (21%), followed by skin & soft tissue (12.6%), gastroenteritis (11%), mucositis (8.2%), porta cath (8.2%) and others (9.7%). No obvious focus of infection was found in 25.3% cases.

A total of 5840 blood cultures were received, off these 1048 (18%) were culture positive. Amongst these, 557 (53%) grew Gram positive bacteria, 442 (42%) grew Gram negative bacteria, 43 (4%) fungi and 6 (1%) anaerobes. Sixty out of 1048 (5.7%) positive blood cultures were polymicrobial. Amongst these 93.4% were positive for two organisms, 5% for three and 1.6% for four organisms. Increasing trend was observed in the isolation rate of gram positive bacteria i.e. from 50% in first study period to 54% in the second one. A little drop in isolation rate of gram negative bacteria was noted i.e. from 43% to 41%. No statistically significant difference for the isolation rate of *Staphylococci, Streptococci *and other gram positive bacteria was found between the two study periods, as shown in Table 1.

**Table 1:** Gram positive bacteria isolated from blood cultures in two halves of study period.

	**(Period 1) Positive blood culture**		**(Period II) Positive blood culture**		
			
**Gram positive Bacteremic isolates**	**n = 104**	**%**	**n = 453**	**%**	**P-value**
*Staphylococcus spp*.	56	53.8	250	55.2	0.804
*Staphylococcus aureus*	14	13.5	43	9.5	0.228
*Streptococcus spp*.	6	5.8	25	5.5	0.920
*Enterococcus spp*.	7	6.7	23	5.1	0.501
*Bacillus spp*.	10	9.6	44	9.7	0.976
*Micrococcus spp*.	3	2.9	17	3.8	0.999 ^#^
*Streptococcus pneumoniae*	2	1.9	16	3.5	0.548 ^#^
*Corynebacterium spp*.	5	4.8	25	5.5	0.772
*Streptococcus pyogenes*	1	1.0	5	1.1	0.999 ^#^
*Nocardia spp*.	0	0.0	1	0.2	0.999 ^#^
Other Gram positive organism	0	0.0	4	0.9	0.999 ^#^

p-values calculated by Chi square test otherwise indicated

# p-value calculated by Fisher exact test

Amongst the gram negative bacteria, *Enterobacteriaceae *remained the predominant group, comprising of 65% and 64% of total gram negative bacteria in the first and second study period respectively as shown in Table 2. *E. coli *was the most frequently isolated organism in both study periods. The isolation frequency of non enterobacteriaceae was found to be static in both study periods i.e. 25–30% of the total gram negative organisms. Amongst this group, *Pseudomonas aeruginosa *was the commonest organism isolated during first study period followed by *Acinetobacter *spp. However, during the second period *Acinetobacter *species was the most frequent pathogen. Another important finding was the emergence of *Stenotrophomonas maltophilia, Aeromonas *species and *Achromobacter *species during the second study period.

**Table 2:** Gram negative bacteria isolated from blood cultures in two halves of study period

	**(Period 1) Positive blood culture**		**(Period II) Positive blood culture**		
			
**Gram Positive Bacteremic Isolates**	**n = 90**	**%**	**n = 352**	**%**	**P-value**
*E. coli*	26	28.9	129	36.6	0.169
*P. aeruginosa*	15	16.7	34	9.7	0.059
*Enterobacter *spp.	12	13.3	30	8.5	0.165
*Pseudomonas *spp.	8	8.9	29	8.2	0.842
*Klebsiella *spp.	11	12.2	41	11.6	0.880
*Acinetobacter *spp.	6	6.7	52	14.8	0.042 *
*Salmonella*	4	4.4	12	3.4	0.751^#^
*Citrobacter *spp.	3	3.3	6	1.7	0.396 ^#^
*Proteus *spp.	3	3.3	1	0.3	0.028 ^#^*
*Aeromonas *spp.	1	1.1	8	2.3	0.693 ^#^
*Stenotrophomonas maltophilia*	0	0.0	7	2.0	0.353 ^#^
*Achromobacter *spp	0	0.0	1	0.3	0.999 ^#^
*Alcaligenes xylosoxidans*	1	1.1	0	0.0	0.204 ^#^
*Chryseomonas luteola*	0	0.0	1	0.3	0.999 ^#^
Campylobacter spp.	0	0.0	1	0.3	0.999 ^#^

p-values calculated by Chi square test otherwise indicated

# p-value calculated by Fisher exact test

* p < 0.05

A significant rise in drug resistant isolates was noted in the second study period. This increase was identified for all gram negative bacteria including *Enterobacteriaceae*, *Pseudomonas aeruginosa*, other *Pseudomonas *species and *Acinetobacter *species as shown in Table 3a &3b. Amongst the *Enterobacteriaceae*, statistically significant resistance appeared in the second study period against ceftriaxone (p = 0.001), ciprofloxacin/ofloxacin (p = 0.003) and piperacillin/tazobactam (p = 0.006). However no resistance against imipenem/meropenem was detected in second study period. Fifty four percent of *Enterobacteriaceae *were ESBL positive during the second study period.

**Table 3:** Difference in sensitivity pattern of frequently isolated Gram negative rods during two halves of study period.

**(a)**										
	**Enterobacteriaceae**					***Pseudomonas. aeruginosa***				
		
	**Period 1**		**Period II**			**Period I**		**Period II**		
						
	**n = 55**	**%**	**n = 212**	**%**	**P-value**	**n = 15**	**%**	**n = 34**	**%**	**P-value**

Amikacin	6	10.9	35	16.5	0.305	0	0	4	11.8	0.298 ^#^
Amox/clav	25	45.5	102	48.1	0.725	NT	NT	NT	NT	-
Ceftriaxone	13	23.6	118	55.7	< 0001*	NT	NT	NT	NT	-
Ciprofloxacin	15	27.3	105	49.5	0.003 *	2	13.3	10	29.4	0.298 ^#^
Tazocin	0	0	24	11.3	0.006 *	0	0	4	11.8	0.298 ^#^
Imipenem	0	0	0	0	-	0	0	2	5.9	0.999 ^#^
Ceftazidime	NT	NT	NT	NT	-	1	6.7	2	5.9	0.999 ^#^


**(b)**										

	***Pseudomonas *species**					***Acinetobactor***				
		
	**Period 1**		**Period II**			**Period 1**		**Period II**		
						
	**n = 8**	**%**	**n = 29**	**%**	**p-value**	**n = 6**	**%**	**n = 52**	**%**	**p-value**

Amikacin	4	50.0	12	41.4	0.705 ^#^	1	16.7	27	51.9	0.195 ^#^
Amox/clavilunate	NT	NT	NT	NT	-	NT	NT	NT	NT	-
Ceftriaxone	0	0	7	24.1	0.308 ^#^	0	0	39	75.0	0.001 ^#^*
Ciprofloxacin	1	12.5	7	24.1	0.655 ^#^	0	0	33	63.5	0.004 ^#^*
Pipracillin/Tazobactam	0	0	2	6.9	0.999 ^#^	0	0	34	65.4	0.003 ^#^*
Imipenem	0	0	6	20.7	0.305 ^#^	0	0	34	65.4	0.003 ^#^*
Ceftazidime	NT	NT	NT	NT	-	NT	NT	NT	NT	-

p-values calculated by Chi square test otherwise indicated

# p-value calculated by Fisher exact test

* p < 0.05

Similarly increased resistance in *Pseudomonas aeruginosa *was noted against amikacin and ciprofloxacin/ofloxacin during the second study period. However, this difference was not statistically significant (p > 0.05). No resistance against piperacillin/tazobactam and imipenem/meropenem was found during the first study half; however resistance against both of these antibiotics (6%) appeared in the second study period. The susceptibility pattern of *Acinetobacter *species shifted from sensitive to highly resistant one with significant p values against third generation cephalosporin (p = 0.001), quinolone (p = 0.004), piperacillin/tazobactam (p = 0.003) and imipenem/meropenem (p = 0.003). During the second study period 37% (49/134) of total non- enterobacteriaceae were found imipenem/meropenem resistant making a total of 14% (49/352) imipenem resistance amongst all gram negative bacteria.

Among the gram positive bacteria isolation frequency of methicillin resistant *Staphylococcus aureus *was not statistically significant (p = 0.524), however, vancomycin resistant *Enterococcus *species emerged in second half of study as shown in Table 4. No *Staphylococcus *species resistant to vancomycin was isolated.

**Table 4:** Difference in sensitivity pattern of frequently isolated Gram positive bacteria during two halves of study period.

	***Staphylococcus *species other than aureus**				***Staphylococcus aureus***						***Enterococcus *species**				
			
	**Period 1**		**Period II**			**Period 1**		**Period II**			**Period 1**		**Period II**		
									
	**n = 56**	**%**	**n = 250**	**%**	**p-value**	**n = 14**	**%**	**n = 43**	**%**	**p-value**	**n = 7**	**%**	**n = 23**	**%**	**p-value**
Vancomycin	0	0	0	0	-	0	0	0	0	-	0	0	3	13.0	0.999 ^#^
Cloxacillin	30	53.6	130	52.0	0.831	3	21.4	13	30.2	0.735 ^#^	NT	-	NT	-	-
Penicillin	49	87.5	192	76.8	0.077	12	85.7	40	93.0	0.587 ^#^	NT	-	NT	-	-
Clindamycin	20	35.7	82	32.8	0.676	3	21.4	13	30.2	0.735 ^#^	NT	-	NT	-	-
Ampicillin	NT	-	NT	-	-	NT	-	-	NT	-	7	100	17	73.9	0.290 ^#^

p-values calculated by Chi square test otherwise indicated

# p-value calculated by Fisher exact test

## Discussion

Our study showed rising trend of carbapenem resistant Gram negative and vancomycin resistant Gram positive bacteria among bacteremic isolates of febrile neutropenic patients. The emergence of carbapenem resistant isolates in our center is likely to be associated with change in antibiotic policies. The two study periods were chosen based on difference in empirical antibiotic choice in our center. During nineties third generation cephalosporin was the empirical antibiotic choice for febrile neutropenic patients at our institute [14]. However, rising trend of resistance against this group of antibiotics was observed among *Enterobacteriaceae *and *Pseudomonas aeruginosa *[15,16]. Prior to year 2000, there was limited availability of piperacillin/tazobactam and imipenem. These agents were introduced throughout the hospital in year 2000 [15].

Our study revealed no resistance to carbapenem and minimal resistance against piperacillin/tazobactam in Gram negative isolates during the first study period. However, rising trend of resistance against these agents was noted in second period. This change was specific for non enterobacteriaceae group. These bacteria showed significant rise in resistance against major antibiotics including third generation cephalosporin, quinolone, amikacin, piperacillin/tazobactam and carbapenem. In contrast to this no resistance against carbapenem was noted amongst *Enterobacteriaceae *group.

Another important finding was increased isolation rates of *Acinetobacter *species. This organism was the sixth Gram negative bacteria isolated during the first study period; however it became the second most commonly isolated gram negative bacteria during the second study period. Moreover, carbapenem resistance in this bacterium was significantly high (p = 0.003) during second study period, indicating possible role of nosocomial transmission for this rising trend. Finally, emergence of Gram negative organism inherently resistant to carbapenem like *Stenotrophomonas maltophilia *during the second study period was another area of concern, a finding consistent with other reports [17]. Our findings are in contrast to other regional reports, where carbapenem resistance in this population is not observed [18,19].

The isolation rate of MRSA during the two study halves was comparable (p = 0.524). The isolation rate of MRSA (31%) was significantly high when compared with the previous study done in 1991 which reported 100% susceptibility against cloxacillin [14]. Similar increase in isolation rate of methicillin resistant *Staphylococcus aureus *(MRSA) was also reported in another study from Taiwan [20]. Moreover emergence of vancomycin resistant *Enterococcus *species (VRE) not only poses a therapeutic challenge for febrile neutropenic patients [21,22] but also indirectly reflects irrational usage of vancomycin and poor infection control practices. The increasing rates of antimicrobial resistance amongst both Gram-positive and Gram-negative pathogens isolated from patients with neutropenia are posturing new therapeutic challenges. These challenges are compounded by the fact that relatively few new drugs are being developed, particularly those that treat resistant Gram-negative organisms [22]. As these trends are often associated with local treatment practices [22,23] therefore, we suggest rational use of broad-spectrum antibiotics especially carbapenem and vancomycin to prevent increasing resistance against them. In addition compromised infection control practice is another contributing factor, hence early detection and prompt isolation of patients with strict compliance to hand hygiene is important to prevent further spread of multi resistant organisms.

## Conclusion

This rising trend of highly resistant organisms stresses the increasing importance of continuous surveillance system and stewardship of antibiotics as strategies in the overall management of patients with febrile neutropenia.

## Competing interests

The authors declare that they have no competing interests.

## Authors' contributions

SI conceived, planned, conducted and generated results of the study, and drafted the manuscript. FI performed data collection and analysis and contributed in manuscript writing. FH and VM performed the statistical analysis and contributed in manuscript writing. SA contributed in provision of clinical information and approved the final manuscript. RH participated in study design and coordination and had contributed in manuscript writing and final approval of script. All authors read and approved the final manuscript.

## Pre-publication history

The pre-publication history for this paper can be accessed here:


